# Supplementary material for: Elucidating Differences in Early-Stage Centrosome Amplification in Primary and Immortalized Mouse Cells
Source: Int J Mol Sci. 2023 Dec 27;25(1):383. doi: 10.3390/ijms25010383 (PMC10778991; doi:10.3390/ijms25010383)
Supplement: Supplementary file 1 [file ijms-25-00383-s001.zip › ijms-2757010-supplementary.pdf]

Supplemental Table S1. **Changes in the levels of proteins related to centrosome duplication 24 h after 200 nM doxorubicin treatment and 10-Gy γ-irradiation to primary MEF and cell line MEF.**

<primary>	Cont./Cont.	D200/Cont. (Mean±S.D.)	p value
p53	1.00<br>-	0.91<br>0.13	0.18
p21	1.00<br>-	2.17<br>0.38	0.02
Skp2	1.00<br>-	2.37<br>0.30	0.01
p27	1.00<br>-	0.31<br>0.09	0.003
NPM	1.00<br>-	0.95<br>0.21	0.36
pNPM 199	1.00<br>-	0.72<br>0.14	0.04
pNPM/NPM	1.00<br>-	0.77<br>0.07	0.01
Tubulin	1.00<br>-	1.00<br>0.00	-

<primary>	Cont./Cont.	10Gy/Cont. (Mean±S.D.)	p value
p53	1.00<br>-	1.45<br>0.22	0.04
p21	1.00<br>-	1.32<br>0.22	0.07
Skp2	1.00<br>-	0.91<br>0.14	0.19
p27	1.00<br>-	0.85<br>0.04	0.01
NPM	1.00<br>-	1.91<br>0.16	0.01
pNPM 199	1.00<br>-	1.44<br>0.13	0.01
pNPM/NPM	1.00<br>-	0.75<br>0.01	0.0001
Tubulin	1.00<br>-	1.00<br>0.00	-

<cell line>	Cont./Cont.	D200/Cont.	p value
p53	1.00<br>-	1.35<br>0.11	0.01
p21	1.00<br>-	1.52<br>0.34	0.06
Skp2	1.00<br>-	4.55<br>1.44	0.03
p27	1.00<br>-	0.46<br>0.13	0.01
NPM	1.00<br>-	1.05<br>0.20	0.34
pNPM 199	1.00<br>-	4.60<br>0.99	0.01
pNPM/NPM	1.00<br>-	4.40<br>0.81	0.01
Tubulin	1.00<br>-	1.00<br>0.00	-

<cell line>	Cont./Cont.	10Gy/Cont.	p value
p53	1.00<br>-	1.43<br>0.21	0.04
p21	1.00<br>-	1.10<br>0.24	0.27
Skp2	1.00<br>-	2.09<br>0.30	0.01
p27	1.00<br>-	0.34<br>0.10	0.004
NPM	1.00<br>-	0.77<br>0.11	0.03
pNPM 199	1.00<br>-	2.10<br>0.40	0.02
pNPM/NPM	1.00<br>-	2.74<br>0.55	0.02
Tubulin	1.00<br>-	1.00<br>0.00	-

The data from three independent experiments are presented. The density of each protein was calculated using ImageJ software and was digitized. The density of each band was normalized to that of α-tubulin, and the ratio of the density of the respective protein treated with doxorubicin or γ-irradiation was divided by that of untreated band and shown as D200/Cont. or 10Gy/Cont., respectively. Mean values, standard deviations, and *p* -values from three experiments are shown.
